# Low Diastolic Blood Pressure Predicts Good Clinical Outcome in Patients With Cerebral Venous Thrombosis

**DOI:** 10.3389/fneur.2021.649573

**Published:** 2021-09-09

**Authors:** Min Li, Liqun Pan, Xiaogang Gao, Jiaojiao Hou, Ran Meng, Xunming Ji

**Affiliations:** ^1^Department of Neurology, Xuanwu Hospital, Capital Medical University, Beijing, China; ^2^Beijing Institute for Brain Disorders, Capital Medical University, Beijing, China; ^3^Department of Medicine, Tianjin Huanhu Hospital, Tianjin, China; ^4^Department of Neurology, Rongcheng City People's Hospital, Baoding, China; ^5^Department of Neurosurgery, Xuanwu Hospital, Capital Medical University, Beijing, China

**Keywords:** blood pressure, intracranial hypertension, severity, prognosis, cerebral venous thrombosis (CVT)

## Abstract

**Background:** Cerebral venous thrombosis (CVT) refers to a stroke subtype characterized by the disturbance of cerebral venous outflow caused by venous thrombosis. Previous studies have reported a range of factors that predict the prognosis of CVT. This study is aimed to find out whether systolic blood pressure (SBP) and diastolic blood pressure (DBP) are suitable as potential indicators of the severity and clinical outcome in CVT patients.

**Methods:** The CVT patients admitted to Xuanwu Hospital from January 2014 to December 2019 were enrolled. The severity of CVT was assessed by the National Institute of Health Stroke Scale (NIHSS) and intracranial pressure (ICP) at the time of admission. The modified Rankin score (mRS) was assessed at 6 months of follow-up.

**Results:** One hundred fifty-six CVT patients were enrolled with a mean age of 35.8 ± 12.8 years. A percentage of 55.8% of the CVT patients recruited were female, and 17.3% were either pregnant or in perinatal period. Headache was the most common symptom. SBP and DBP were not correlated with NIHSS at admission. Furthermore, SBP and DBP had no impact on the disturbance of consciousness, epilepsy, intracranial hemorrhage, and mental disorders. However, SBP and DBP were positively correlated with ICP at admission. SBP > 129.5 mmHg and/or DBP > 77.5 mmHg suggested the presence of intracranial hypertension (IH). Based on current results, SBP was not correlated with mRS at 6 months of follow-up. However, DBP was found to be positively correlated with mRS at 6 months of follow-up. DBP in CVT patients with good prognosis was significantly lower than in those with poor prognosis. DBP > 79.5 mmHg was identified as a cutoff value to predict a poor clinical outcome. A higher mRS and a higher rate of poor clinical outcome were found in CVT patients with SBP > 146 mmHg or DBP > 79.5 mmHg compared to those with SBP ≤ 146 mmHg or DBP ≤ 79.5 mmHg.

**Conclusion:** SBP > 129.5 mmHg and DBP > 77.5 mmHg suggested the presence of IH in CVT patients. DBP > 79.5 mmHg predicted a poor clinical outcome.

## Introduction

Cerebral venous thrombosis (CVT) refers to a stroke subtype characterized by the cerebral venous outflow disturbance caused by venous thrombosis (1). In recent studies, it has been demonstrated that the incidence of CVT is potentially higher than expected, reaching 1.32–1.57/100,000 people annually (2). The clinical manifestations of CVT are highly unpredictable (2). In severe CVT cases, patients suffered from disturbance of consciousness, new onset of epilepsy, intracranial hemorrhage, and mental disorders (3, 4).

It is reported that 13.4% of CVT patients had poor prognosis (5). Also, a series of studies have revealed that the following factors predicted poor clinical outcome: male, older age, an increase in National Institutes of Health Stroke Scale (NIHSS) ≥3 at admission, bilateral motor signs, malignancy, central nervous system infection, coma, mental disorders, deep cerebral venous thrombosis, hemorrhagic infarcts, and midline shift (5–7).

It is widely recognized that high blood pressure (BP) is a significant risk factor for arterial stroke and an indicator of poor prognosis in arterial stroke patients (8). However, there are few studies focusing on the role of blood pressure in CVT. The aim of this study is to investigate whether systolic blood pressure (SBP) and diastolic blood pressure (DBP) can be applied as potential indicators of severity and clinical outcome for CVT patients.

## Methods

### Subject Recruitment

The CVT patients admitted to Xuanwu Hospital from January 2014 to December 2019 were enrolled. This prospective study was approved by the Xuanwu Hospital ethnics committee. The inclusion criterion was CVT confirmed by magnetic resonance venography (MRV), computed tomography venography (CTV), digital subtraction angiography (DSA), or high resolution-magnetic resonance imaging (HR-MRI).

Intracranial pressure (ICP) was detected by measuring CSF pressure with lumbar puncture. An ICP > 250 mmH_2_O was considered as intracranial hypertension (IH) (9). In order to evaluate the clinical outcome of CVT, the modified Rankin score (mRS) was assessed at 6 months of follow-up. mRS ≤ 2 and mRS > 2 were treated as good clinical outcome and poor clinical outcome, respectively (10).

### Statistical Analysis

SPSS Version 16.0 (SPSS, Inc., Chicago, IL, USA) was used for all statistical analyses. The continuous data following Gaussian distribution were expressed as mean ± standard deviation and analyzed with independent *t*-test, while categorical data were expressed as a number (percentage) and processed using chi-square test. Pearson correlation coefficient and linear regression were used to predict the correlation between continuous variables. Logistic regression models were constructed using enter method. The cutoff points were calculated using receiver operating characteristic (ROC) curves. *p* < 0.05 was considered statistically significant.

## Results

### Baseline Demographic Features of CVT Patients

One hundred fifty-six CVT patients were recruited in this study, with a mean age of 35.8 ± 12.8 years. There were 87 (55.8%) female patients, and 27 (17.3%) patients were either pregnant or in the perinatal period. Twenty-one (13.5%) patients had hypertension, 7 (4.5%) had diabetes mellitus, 7 (4.5%) had hyperlipidemia, 4 (2.6%) had deep venous thrombosis, 4 (2.6%) had pulmonary embolism, and 3 (1.9%) had systemic infectious diseases. Seventeen (10.9%) patients had a history of smoking, and 17 (10.9%) patients had a history of alcohol. Headache (89.7%) was the most frequent symptom, followed by new-onset epilepsy (34.6%), limb weakness (33.3%), disturbance of consciousness (21.2%), visual impairment (19.9%), and tinnitus (5.8%). The average SBP and DBP were 122.8 ± 16.7 and 77.6 ± 10.8 mmHg, respectively (Table 1).

**Table 1:** Baseline demographic features and clinical characteristics of patients with CVT.

**Characteristics**	**Enrolled subjects (*N* = 156)**
Age (years)	35.8 ± 12.8
Female	87 (55.8%)
Pregnancy or perinatal period	27 (17.3%)
**Co-morbid disease**	
Hypertension	21 (13.5%)
Diabetes mellitus	7 (4.5%)
Hyperlipidemia	7 (4.5%)
Deep venous thrombosis	4 (2.6%)
Pulmonary embolism	4 (2.6%)
Systemic infectious disease	3 (1.9%)
Smoking	17 (10.9%)
Alcohol	17 (10.9%)
**Clinical manifestations**	
Headache	140 (89.7%)
Visual impairment	31 (19.9%)
Tinnitus	9 (5.8%)
Disturbance of consciousness	33 (21.2%)
New-onset epilepsy	54 (34.6%)
Limb weakness	52 (33.3%)
**Blood pressure at admission**	
Systolic pressure (mmHg)	122.8 ± 16.7
Diastolic pressure (mmHg)	77.6 ± 10.8

### Blood Pressure Correlates With the Severity of CVT

**Figure 1:**
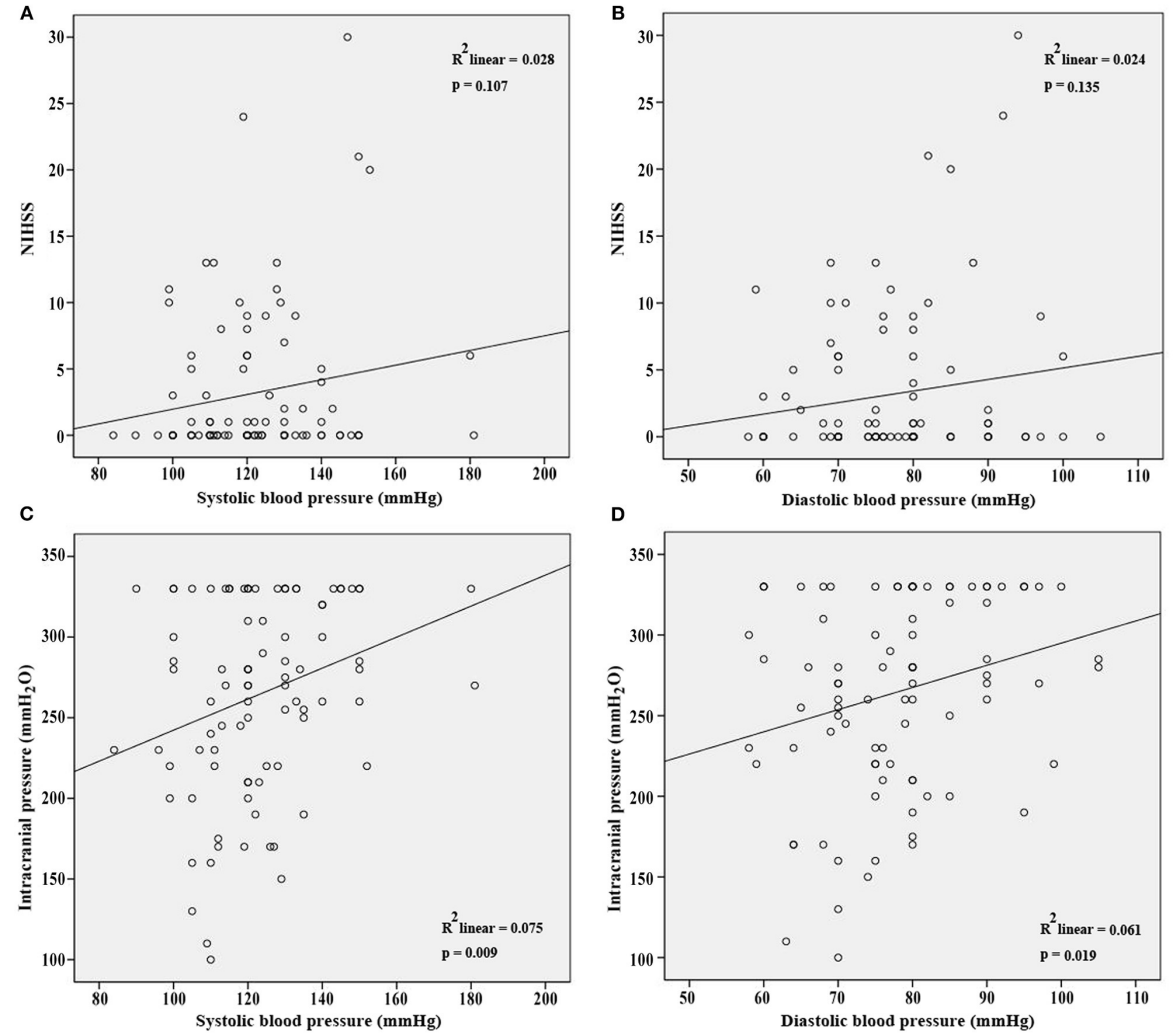
SBP and DBP were not correlated with NIHSS **(A,B)** at admission. However, SBP and DBP were positively correlated with ICP at admission **(C,D)**.

**Figure 2:**
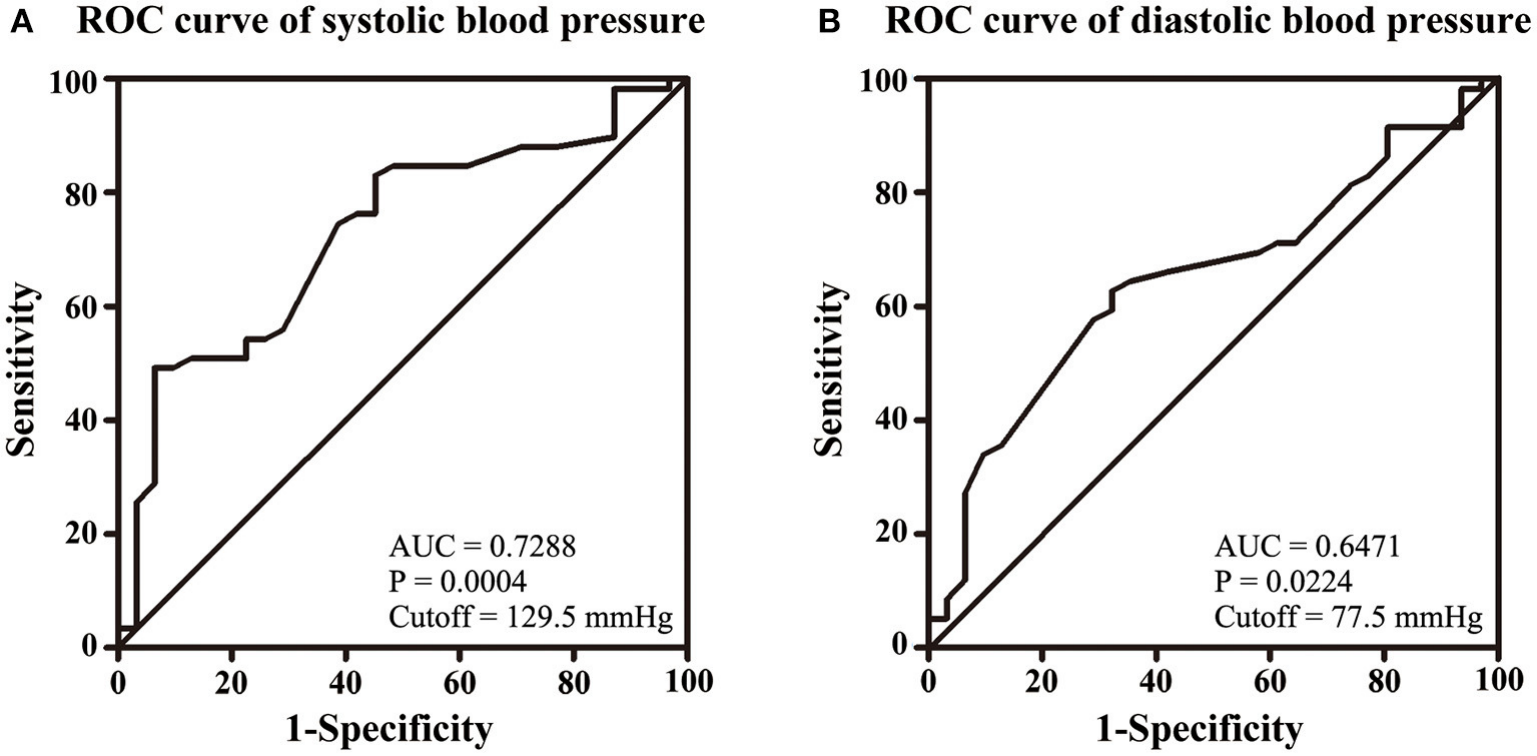
Volumes of 129.5 mmHg of SBP **(A)** and 77.5 mmHg of DBP **(B)** were identified as cutoff values for IH.

NIHSS, clinical presentations, and ICP at admission were used to assess the severity of CVT. Our results showed that SBP and DBP were not correlated with NIHSS (*p* = 0.107; *p* = 0.135, Figures 1A,B). Results from the present study also revealed that SBP and DBP had no impact on the disturbance of consciousness, epilepsy, intracranial hemorrhage, and mental disorders (Table 2). However, both SBP and DBP were positively correlated with ICP (*p* = 0.009; *p* = 0.019, Figures 1C,D). The ROC curve was used to identify the cutoff values of SBP and DBP. SBP > 129.5 mmHg (AUC = 0.7288, *p* = 0.0004, Figure 2A) and DBP > 77.5 mmHg (AUC = 0.6471, *p* = 0.024, Figure 2B) were identified as indicators for IH in CVT patients.

**Table 2:** Both SBP and DBP had no impact on disturbance of consciousness, epilepsy, intracranial hemorrhage and mental disorders.

**Independent variables**	**Dependent variables**	**OR**	***P***	**Confidence interval**	
				**Lower**	**Upper**
SBP	Conscious disturbance	0.979	0.131	0.953	1.006
	Epilepsy	1.013	0.266	0.990	1.036
	Intracranial hemorrhage	1.010	0.508	0.981	1.040
	Mental disorder	1.010	0.417	0.986	1.036
DBP	Conscious disturbance	0.989	0.578	0.951	1.029
	Epilepsy	1.013	0.465	0.978	1.049
	Intracranial hemorrhage	0.996	0.869	0.951	1.044
	Mental disorder	1.008	0.694	0.970	1.047

### DBP Predicts the Prognosis of CVT

Subsequently, we aimed to evaluate the impact of BP on the prognosis of CVT. SBP was not correlated with mRS at 6 months of follow-up after adjusting for ICP and DBP (*p* = 0.784; *p* = 0.159, Table 3). Notably, DBP was positively correlated with mRS at 6 months of follow-up after adjusting for ICP and SBP (*p* = 0.039, Table 3).

**Table 3:** Neither SBP nor ICP was correlated with mRS at 6 months of follow-up.

**Independent**	**Dependent**	**Coeff (B)**	**Std Coeff (β)**	**Significance (P)**
**variables**	**variables**			
**mRS at 6 months of follow-up**				
SBP	mRS	0.003	0.010	0.784
DBP		0.027	0.016	0.039^*^
ICP		0.015	0.011	0.159

*However, DBP was positively correlated with mRS at 6 months of follow-up*.

* *p < 0.05*.

**Figure 3:**
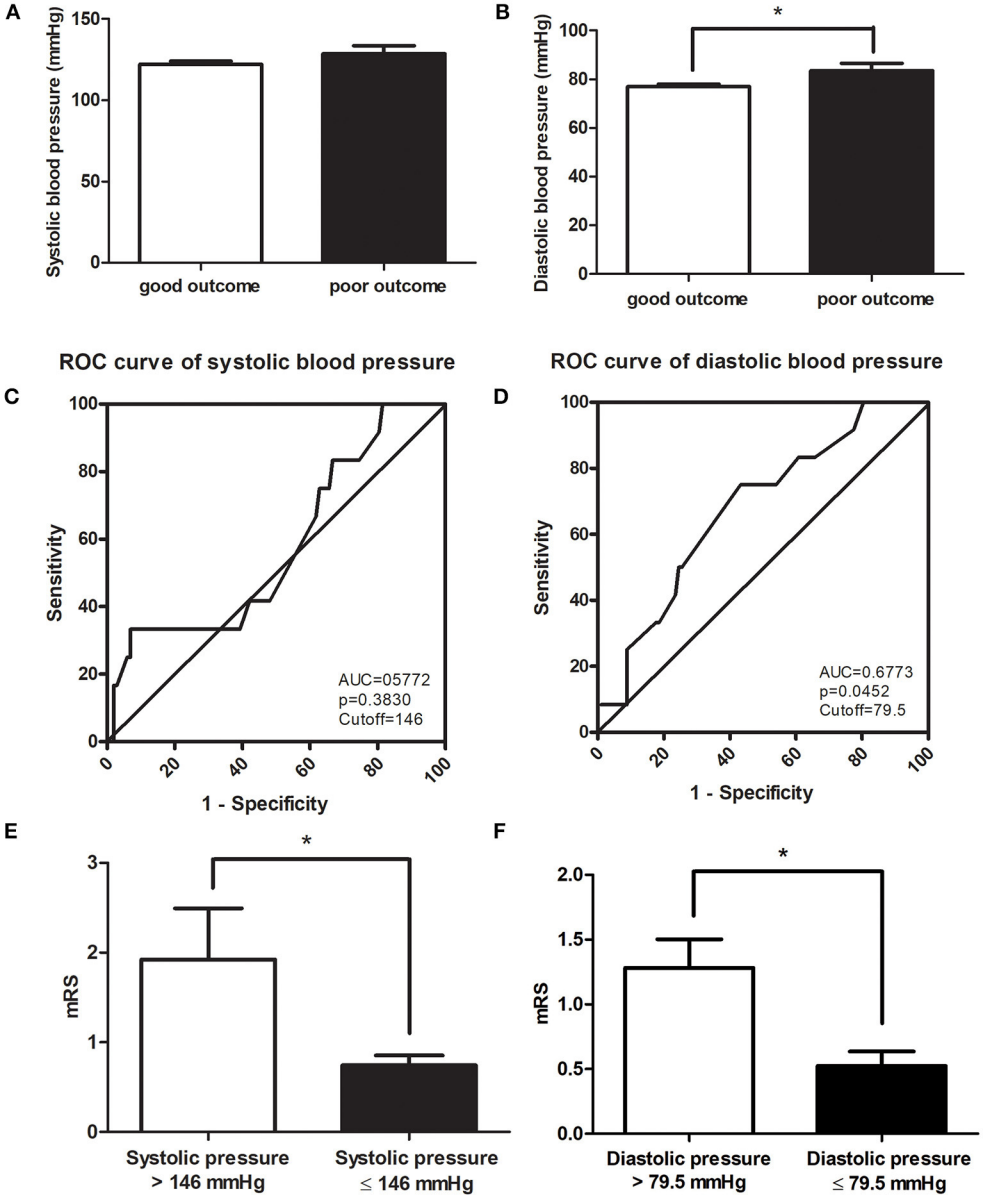
No significant difference was found in SBP between CVT patients with good prognosis and those with poor prognosis **(A)**. DBP in CVT patients with good prognosis was significantly lower than in those with poor prognosis **(B)**. A volume of 146 mmHg of SBP as a cutoff value exhibited low sensitivity and specificity **(C)** whereas DBP < 79.5 mmHg predicted a good clinical outcome with high sensitivity and specificity **(D)**. CVT patients with SBP > 146 **(E)** or DBP > 79.5 mmHg **(F)** had significantly higher mRS compared to those with SBP ≤ 146 mmHg **(E)** or DBP ≤ 79.5 mmHg **(F)**. *p < 0.05.

No significant difference was found in SBP between the CVT patients with good prognosis and those with poor prognosis (*p* = 0.222, Figure 3A). DBP in the subjects with good prognosis was significantly lower compared with those with poor prognosis (*p* = 0.046, Figure 3B). SBP of 146 mmHg and DBP of 79.5 mmHg were identified as the cutoff values (Figures 3C,D). However, SBP 146 mmHg as a cutoff value exhibited low sensitivity and specificity (AUC = 0.5772, *p* = 0.3830, Figure 3C). DBP > 79.5 mmHg predicted a poor clinical outcome in CVT patients (AUC = 0.6733, *p* = 0.0452, Figure 3D). CVT patients with SBP > 146 mmHg or DBP > 79.5 mmHg had significantly higher mRS compared to those with SBP ≤ 146 mmHg or DBP ≤ 79.5 mmHg (*p* = 0.002, *p* = 0.002, Figures 3E,F). A higher rate of poor clinical outcome was found in CVT patients with SBP > 146 mmHg or DBP > 79.5 mmHg compared to those with SBP ≤ 146 mmHg or DBP ≤ 79.5 mmHg (*p* = 0.008, *p* = 0.005, Table 4).

**Table 4:** A higher rate of poor clinical outcome was found in CVT patients with SBP > 146 mmHg or DBP > 79.5 mmHg compared to those with SBP ≤ 146 mmHg or DBP ≤ 79.5 mmHg.

		**Good clinical outcome**	**Poor clinical outcome**	**Total**	***P***
SBP	SBP > 146 mmHg	12	6	18	0.008^*^
	SBP ≤ 146 mmHg	126	12	138	
	Total	138	18	156	
DBP	DBP > 79.5 mmHg	58	14	72	0.005^*^
	SBP ≤ 79.5 mmHg	80	4	84	
	Total	138	18	156	

* *p < 0.05*.

## Discussion

A Turkish multicenter study reported that females are more susceptible to CVT (7). Sixty-eight percent of the CVT patients were female. Results from the present study reported a relatively low proportion of female. Only 55.8% of the CVT patients were female. Headache was the most frequent symptom based on results from this study. This finding is consistent with the previous reports (7, 11). The limitation of this study was the small sample size. For stronger evidence, a multicenter study recruiting CVT patients from 25 tertiary hospitals across China Mainland led by our research team is ongoing (NCT 03919305). Results from this multicenter study will be published later.

Our results also revealed that SBP and DBP were not correlated with NIHSS at admission. However, the results were not strong enough to draw a final conclusion that SBP and DBP were not correlated with the severity of CVT. NIHSS was designed to evaluate the severity of arterial stroke (12). It showed low sensitivity and specificity for CVT. For example, a CVT patient with headache and seizures was scored as 0. The severity of the clinical presentations was currently discussed more descriptively. Yet, there was no severity evaluation scale for CVT patients based on their clinical presentations. Thus, a modified NIHSS for venous stroke is required.

SBP and DBP were found to be positively correlated with ICP. It is suggested that higher SBP and DBP would ensure the perfusion pressure in the presence of IH among CVT patients (13). It seems contradictory that SBP and DBP were positively correlated with ICP, but had no impact on the disturbance of consciousness, epilepsy, intracranial hemorrhage, and mental disorders. Except for IH, inflammation as well as oxidative stress, apoptosis, glutamate excitotoxicity, and dysfunction of the blood–brain barrier were also involved in the pathogenesis of disturbance of consciousness, epilepsy, intracranial hemorrhage, and mental disorders (14–16). The increasing ICP level may not be the determining factor of these clinical manifestations.

In a study conducted by de Bruijn et al. (17), it was reported that CVT patients with IH did not suffer from poor prognosis. Our results also indicated that ICP was not correlated with mRS at 6 months of follow-up after adjusting for SBP and DBP. Although CVT patients with SBP > 146 mmHg had a higher rate of poor prognosis and higher mRS, a cutoff value of 146 mmHg exhibited low sensitivity and specificity. SBP was not correlated with mRS at 6 months of follow-up after adjusting for ICP and SBP. A higher rate of poor prognosis and higher mRS were also observed in CVT patients with DBP > 79.5 mmHg. DBP was positively correlated with mRS at 6 months of follow-up after adjusted for SBP and ICP. DBP > 79.5 mmHg suggested a poor prognosis with high sensitivity and specificity.

The explanations for this observation were as follows: first, increased DBP may be due to a markedly elevated ICP which results in aggravation of inflammation, oxidative stress, glutamate excitotoxicity, and dysfunction of the blood–brain barrier (14–16). Second, a number of studies suggested that elevated venous pressure promoted the contraction of arteriolar smooth muscle, thus increasing the peripheral vascular resistance. This may further lead to elevated DBP (18, 19). A higher level of DBP may be correlated with an elevated central venous pressure which reduces the cerebral venous return. However, this hypothesis requires verification with further investigations.

## Conclusions

Both SBP and DBP were positively correlated with ICP at admission. SBP > 129.5 mmHg and DBP > 77.5 mmHg suggested the presence of IH in CVT patients. DBP was positively correlated with mRS at 6 months of follow-up. DBP > 79.5 mmHg predicted a poor clinical outcome in CVT patients.

## Data Availability Statement

The raw data supporting the conclusions of this article will be made available by the authors, without undue reservation.

## Ethics Statement

The studies involving human participants were reviewed and approved by the ethics board of Xuanwu Hospital. Written informed consent to participate in this study was provided by the participants' legal guardian/next of kin.

## Author Contributions

ML: contributed to study design and drafting the manuscript. LP: contributed to study design and data collection. XG: contributed to data analysis. JH: contributed to data collection. RM: contributed to acquisition of study funding, study design, and critical revision of the manuscript. XJ: contributed to data interpretation and critical revision of the manuscript. All authors contributed to the article and approved the submitted version.

## Funding

This study was supported by the National Key R&D Program (2017YFC1308401), the National Natural Science Foundation (81371289) and the Project of Beijing Municipal Top Talent of Healthy Work of China. This study was also funded by the Natural Science Foundation of Beijing Municipality (7212047). The grant number of Project of Beijing Municipal Top Talent of Healthy Work is 2014-2-015.

## Conflict of Interest

The authors declare that the research was conducted in the absence of any commercial or financial relationships that could be construed as a potential conflict of interest.

## Publisher's Note

All claims expressed in this article are solely those of the authors and do not necessarily represent those of their affiliated organizations, or those of the publisher, the editors and the reviewers. Any product that may be evaluated in this article, or claim that may be made by its manufacturer, is not guaranteed or endorsed by the publisher.
